# Tropical Laboratory Initiative: An innovative model for laboratory medicine in rural areas

**DOI:** 10.4102/ajlm.v8i1.922

**Published:** 2019-09-26

**Authors:** Zelda R. Moran, Atta B. Frimpong, Pablo Castañeda-Casado, Francis K. Frimpong, Manuela B. de Lorenzo, Yanis Ben Amor

**Affiliations:** 1 Earth Institute, Columbia University, New York, New York, United States; 2 Millenium Promise Alliance, Accra, Ghana; 3 GlaxoSmithKline, Brentford, United Kingdom; 4 Center for Sustainable Development, Earth Institute, Columbia University, New York, New York, United States

**Keywords:** laboratory, burden of disease, community health, data quality, disease reporting, epidemiology, global health, health services, maternal and child health, rural health

## Abstract

**Background:**

Communities in rural, low-resource settings often lack access to reliable diagnostics. This leads to missed and misdiagnosed cases of disease and contributes to morbidity and mortality.

**Objective:**

This paper describes a model for providing local laboratory services to rural areas of Ghana, and provides suggestions on how it could be adapted and expanded to serve populations in a range of rural communities.

**Methods:**

The Tropical Laboratory Initiative (TLI) system in Ghana comprises one central laboratory where samples delivered from clinics by motorbike riders are analysed. Test requests and results are communicated on a mHealth application, and the patient does not have to visit the laboratory or travel beyond the clinic to receive a test. The TLI also serves as a research base. The laboratory is accredited by the National Health Insurance Authority, and accepts the national health insurance. The TLI serves several communities in Amansie West, Ashanti region, and currently works with 10 clinics. The nearest hospital is a one-hour drive away and is the only other nearby facility for diagnostics beyond basic rapid tests.

**Results:**

Demand for services has increased yearly since the launch in 2010, and the TLI currently provides over 1000 tests to approximately 350 patients monthly. The majority of patients are female, and the most common tests are for antenatal care. Our experience demonstrates that laboratory services can be affordable and most components already exist, even in rural areas.

**Conclusion:**

Ministries of health in low-resource settings should consider this model to complement the rapid tests available in clinics. Integrating with an insurance system promotes financial sustainability.

## Introduction

Access to reliable diagnostics can be a barrier to appropriate, timely medical treatment in resource-limited settings. Under-diagnosis and misdiagnosis of diseases contribute to morbidity and mortality, often despite the existence of effective treatments. Primary care laboratory medicine can help address this gap, and should be a priority for health system strengthening programmes.^1,2,3^ Access to diagnostics in rural, low-resource settings is often either nonexistent or limited to basic rapid diagnostic tests, forcing healthcare providers to rely on referral or presumptive treatment. Here, we present results and reflection on a rural laboratory network in Ghana called the Tropical Laboratory Initiative (TLI), a collaboration between Columbia University, Ghana Health Service, Millennium Promise Alliance, and Ghana’s National Health Insurance Authority. Our model could be used as a template for increasing access to laboratory services in low-resource rural communities worldwide.

Several examples of rural diagnostic laboratory and specimen referral systems have been described in sub-Saharan Africa, the Caribbean, and Vietnam.^4^ In Uganda, a specimen referral system consisting of ‘hub’ laboratories around the country to serve rural ‘spoke’ health facilities was first initiated in 2013,^5^ and samples are transported to each hub by motorbike – a model quite similar to the one described here.^4,5,6,7^ In Haiti, a similar hub and spoke model has been implemented since 2011 but with a focus on improving access to HIV testing and monitoring at laboratories with CD4+ count and viral load testing services.^8^ In Ethiopia, a referral system for blood samples and dried blood spots utilises both couriers and the national postal system for transporting specimens from lower-tiered to higher-tiered health facilities with more advanced laboratory services.^4,9^ Publications on these initiatives are scarce, but the results observed include reduced turn-around time in Ethiopia and Uganda, and reduced cost of sample processing in Uganda.^4,7^ The aim of the TLI is to establish a model of specimen referral and increased access to diagnostic testing for rural populations in Ghana, with an added element of same-day delivery of electronic test results to healthcare providers, and integration with the National Health Insurance Scheme (NHIS) to support financial sustainability.

## Purpose

The TLI was established in the south-western Ashanti region in 2010 with the goal of improving the quality of and access to diagnostics for a network of neighbouring clinics. Prior to the inception of the TLI, none of the clinics offered diagnostics beyond basic rapid diagnostic tests, which do not exist for many common diseases and health conditions affecting the population. As a result, most patients were referred to the nearest district hospital for testing, about a one-hour drive from the referring clinics. People were often unable to make this journey, especially if they were ill or had trouble accessing transportation. In addition, it is possible that the community may have lost confidence in local clinics and bypassed them completely, going directly to the district hospital for treatment (authors’ observations). This would result in unnecessary demand for basic testing services at the hospital, and does not promote a strong community-level primary care system. The TLI was created to prevent unnecessary referrals, increase the speed of urgent referrals, and to provide insight into the health needs and disease burdens of rural populations. As of 2019, the TLI provides analysis of blood, urine, stool, and sputum samples from 10 health centres that are 14–45 km away from the nearest hospital (average 30 km), and serve about 31 000 people in several villages. Test results are produced on the same day that samples are received at the laboratory, and often on the same day that the patient presents to the clinic.

### Ethical considerations

This work was approved by the Columbia University Institutional Review Board, number IRB-AAAP3007, as a non-human subjects study.

## Methods

### The Tropical Laboratory Initiative model

The TLI was designed to serve a network of partner clinics. The model consists of one central rural laboratory, composed of three rooms – the main laboratory, a phlebotomy room, and an office – and staffed with two laboratory technologists and two motorbike riders who deliver samples from nearby clinics. The motorbike riders, who are not laboratory scientists, were trained in safe blood collection and sample storage as part of a special training module developed by the President’s Emergency Plan for AIDS Relief (PEPFAR). They support nurses by collecting blood or other specimens from patients as requested by clinic staff. The riders have daily routes during which they visit each clinic three times per week, and remain at each clinic to collect samples for approximately 2 hours. The timing of each route is designed such that the riders are at the clinics on busy days and at times when most patients normally present to the clinic.

**FIGURE 1:**
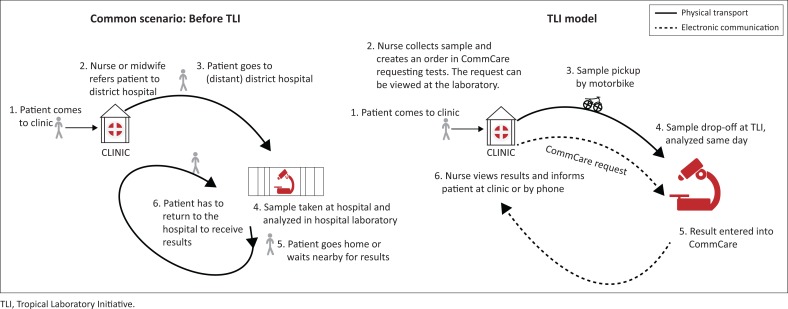
The Tropical Laboratory Initiative model.

The samples are stored at 4 degrees Celsius in refrigerators at the clinics, if they are collected before motorbike riders arrive or on a day when they are not scheduled to come, and are transported in cold chain in insulated Styrofoam to the laboratory. At the beginning of the project in 2010, the TLI served six clinics that received test results on paper forms, but the model has since evolved: paper records were replaced by SMS in 2012, and updated in 2015 to the current tablet-based platform, which works using a mHealth application called CommCare (Dimagi, Cambridge, Massachusetts, United States; https://www.dimagi.com/commcare/). The mHealth system provides forms for test requests, results, and secure data storage, and allows health workers to order tests and view results instantly, while laboratory technologists can view requests and input results onto a tablet. Test results are normally available within 6–12 h of sample collection, or within 48 hours in the case of samples collected when there was no motorbike pickup that day. Results can be viewed by the referring nurse or midwife immediately after they are entered (Figure 1).

Apart from providing community diagnostic services, the laboratory also serves as a research centre for epidemiologic studies and validation of diagnostic devices. An external quality assurance system for haemoglobin, tuberculosis and malaria testing is in place with the affiliated district hospital; samples are tested at the TLI, and sent to the hospital for the same tests. The results are compared to ensure that the TLI test results are of high quality.

### Administrative status

In 2017, the laboratory was accredited as an independent facility by the National Health Insurance Authority, becoming – to our knowledge – the first non-hospital affiliated laboratory serving a rural area in Ghana. Normally, a rural laboratory would be accredited as part of a healthcare facility, which means that payments to the laboratory would go directly to the affiliated clinic or hospital. Independent status enables the laboratory to easily serve patients from multiple facilities, making it easier to provide diagnostics to a wider catchment area.

As a result, the TLI receives insurance reimbursements, so that any Ghanaian enrolled in the NHIS can receive TLI services for free. This supports the sustainability of the business model and promotes universal access to diagnostic services. Uninsured patients pay at rates determined by the NHIS for private laboratories. The TLI will pursue accreditation through Stepwise Laboratory Quality Improvement Process Towards Accreditation (http://www.aslm.org/what-we-do/slipta/) as recommended by the Ghanaian government.

### Diagnostic tests package

As the first laboratory of its kind in Ghana, accreditation involved negotiations with Ghana Health Services and the National Health Insurance Authority on what diagnostics package to offer, and what tests would be inapplicable or impractical at the rural level. A list of services was finalised (Table 1), with the goal of providing diagnostics well beyond what is normally available at the clinic level. Tests for both infectious and chronic diseases, as well as blood and urine analysis for antenatal and postnatal care, are available, allowing clinic health workers to order tests previously unavailable to them, and to request confirmation if there is doubt about any clinic test. While the services offered are greatly expanded compared to the status quo, they are limited to those tests that local clinic health workers are qualified to order and interpret, and that do not require advanced equipment or specialised reagents. As a result, the TLI model could be adapted or replicated in many rural settings, without the need for expensive materials or additional training for health workers.

**TABLE 1:** Diagnostic tests offered at the Tropical Laboratory Initiative site in Tontokrom, Ghana, July 2019.

Test offered	Method used at the Tropical Laboratory Initiative
Malaria	Smear microscopy, used as a confirmatory test after using a rapid diagnostic test at the clinic, since the stained blood film is critical for malaria diagnosis.
White blood cells	Blood is diluted with stained acetic acid, which lyses erythrocytes but not leucocytes. The diluting fluids also contain a dye that stains nuclei. Requires tally counter and Neubauer counting chamber.
Typhoid	*Salmonella* Typhi and *Salmonella* Paratyphi detection by Widal serological test. Requires refrigerator to keep reagents at 2 °C – 8 °C.
Fasting/random blood sugar	Glucose test using a glucometer.
Erythrocyte sedimentation rate	Westergen method. Measures the sedimentation of erythrocytes in their native place. The numerical value is obtained by measuring the distance between the lowest point of the surface meniscus to the upper limit of the erythrocyte sediment in a column of anticoagulated and diluted blood that has stood in the selected tube for 60 minutes. Requires Westergen standard tubes and 3.8% trisodium citrate.
Urine and stool examinations	Detection of protein, blood, leucocytes, parasitic infections in stool, and white blood cells, red blood cells, casts, epithelial cells, bacteria and other cellular components in urine. Requires centrifuge and urinalysis reagent kits.
Pregnancy	Rapid diagnostic test
Tuberculosis	Acid fast bacilli smear microscopy. Requires bunsen burner, biological safety cabinet (if required by local guidelines) or other safety precautions, 20% – 25% sulphuric acid, hydrosoluble methylene blue, and strong carbol fuchsin.
HIV infection	Rapid Diagnostic test
Sickle cell	Oxygen reduction method. Requires sodium metabisulphite.
Blood group and rhesus	Agglutination method
Hepatitis B	Rapid diagnostic test
Haemoglobin	Cyanmethaemoglobin method. Requires colorimeter, drabkins solution, and blood samples of known haemoglobin concentration. Stable electrical power is required
Syphilis (performed when clinics have stock-outs of rapid diagnostics)	Rapid diagnostic test
Sonography	Ultrasound
Glucose-6-phosphate dehydrogenase (G6PD)	Haemoglobin is oxidised to methaemoglobin by sodium nitrite. The redox dye, methylene blue activates the pentose phosphate pathway, resulting in the enzymatic conversion of methaemoglobin back to haemoglobin in those red blood cells with normal (G6PD) activity. In G6PD deficient cells there is no enzymatic reconversion to haemoglobin. Requires water bath, which must be heated continuously to 37 °C.

### Technical and scientific constraints

Operating a rural laboratory implies technological and operational limitations, and the TLI is often affected by power outages, water shortages, and severe seasonal dust. Equipment repairs are more difficult due to the remote location, and certain laboratory materials and reagents can be a challenge to replace during stock-outs. Therefore, all services are provided using low-tech, affordable instruments and accessible reagents, and a power generator is available during outages. All tests are carried out with basic equipment: mainly microscope, centrifuge, water bath, colorimeter, solar-powered refrigerator, and generator (Table 1). One of the main advantages of the model is that centralisation allows for easier supervision: equipment in need of repair is identified immediately and repaired within days, whereas it would be more difficult to address these issues at multiple individual clinics.

## Outcomes

### Sample volume

**FIGURE 2:**
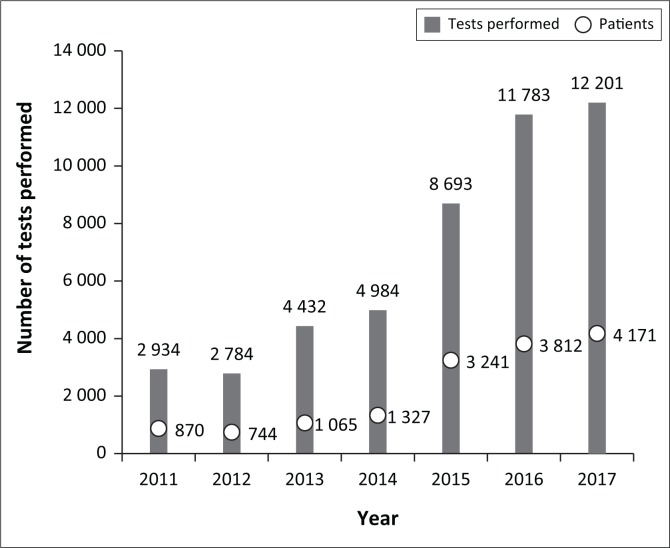
Tests performed at the Tropical Laboratory Initiative by year.

The TLI laboratory was opened in 2010, and the number of tests and patients has increased steadily since 2012 (Figure 2). On average, the number of patient visits has increased by 43% each year, with a 144% increase between 2014 and 2015. In 2017, the TLI served 4171 patients, providing an average of 1019 tests to 350 patients per month. The number of tests requested is highest on days when women normally come for antenatal care, suggesting that this model may be particularly suitable for supporting maternal and child health efforts. Increasing demand suggests that the laboratory is not yet operating at capacity, and has the potential to serve a greater number of people in the surrounding community.

### Patient population

Between 2010 and 2017, the TLI returned test results for 15 247 patient visits and approximately 46 000 individual tests (most visits result in multiple tests), many of which were for pregnant women. Indeed, 82% of all patient visits between 2011 and 2017 were by female patients, and 57% of female patients (47% of all patients) were pregnant. About 42% of visits from male patients were boys under 5 years old, compared to 9% of visits from female patients (Table 2). The majority of female patients (75%) were between 15 and 35 years old. Data on sex was missing for 73 patients (0.48%), and age was missing for 216 (1.42%) patients. (Since the TLI now accepts the national health insurance, it would be possible to determine how often patients have multiple sets of tests ordered by a clinic, if the NHIS number was used as a unique identifier. This could be particularly useful for monitoring how often pregnant women return for antenatal care, and how many women receive the recommended tests, an important indicator for research in maternal and reproductive health.

**TABLE 2:** Demographic data of patients served at the Tropical Laboratory Initiative, Tontokrom, Ghana, 2010–2017.

Variable	Male	Female	Total
**Overall**			
n	2725	12439	15237
%	17.88	81.64	-
**Pregnant**			
n	N/A	7114	7114
%	N/A	57.19	46.69
**Mean Age (Range)**	18.42 (0-92)	24 (0-103)	43.57
**Under 5 yrs (%)**			
n	1141	1137	2278
%	41.87	9.14	14.95

N/A, not applicable; yrs, years.

### Tests performed

Diagnostic services include tests for malaria, typhoid, HIV, and tuberculosis, along with antenatal care bloodwork and stool and urine examination (Table 1, Table 3). Haemoglobin, blood group and rhesus, G6PD deficiency, hepatitis B, and sickle cell (all antenatal tests) compose 67% of tests performed at the TLI. Since 2010, 22% of tests were for haemoglobin, 15% for blood group and rhesus factor, 15% for hepatitis B, 15% for sickle cell, and 7% for syphilis. About 14% of all tests were malaria films, which nurses may request if they suspect malaria but the rapid diagnostic test at the clinic was negative. The remaining tests were for typhoid, parasites (urine and stool), and a limited number of HIV tests (usually performed at the clinics). Overall, 57% of female patients and 8% of male patients were tested for sickle cell disease or its trait: 12% of female patients and 17% of male patients tested positive. These results provide new insights into the disease burdens of the local community, and long-term surveillance could be useful in early detection of outbreaks (in the case of infectious diseases) and for monitoring the success of public health programmes.

**TABLE 3:** Summary of test types performed at the Tropical Laboratory Initiative facility, Tontokrom, Ghana, 2010–2017.

Test type	Percentage of all tests
Haemoglobin	22
Blood Group and Rhesus	15
Hepatitis B	15
Sickle Cell Trait or Disease	15
Malaria Film	14
Syphilis (VDRL)	7
Typhus	5
Urinalysis	4
Stool analysis	1
Other	2

VDRL, Venereal Disease Research Laboratory.

### Infectious disease test results

Many of the infectious disease diagnostics performed at the TLI were also associated with antenatal care, and included syphilis, hepatitis B, and malaria. Among female patients, 8.5% of tests for syphilis and 10% of tests for hepatitis B were positive (Table 4), which is low compared to the estimated 13.1% regional prevalence in Ashanti.^10^

**TABLE 4:** Results of common tests for infectious diseases, Tontokrom, Ghana 2010–2017.

Variable	Sex	Positive	%	Negative	%	Total Tests
Syphilis	F	277	8.47	2992	91.53	3269
	M	28	22.58	96	77.42	124
	**Total**	305	8.99	3088	91.01	3393
Hepatitis B	F	641	9.86	5862	90.14	6503
	M	63	15.40	346	84.60	409
	**Total**	704	10.19	6208	89.81	6912
Malaria	F	2918	60.94	1870	39.06	4788
	M	1456	70.24	617	29.76	2073
	**Total**	4374	63.75	2487	36.25	6861

F, female, M, male.

HIV tests are performed at the TLI only if clinics run out of rapid diagnostic tests, or for confirmation when requested. HIV test results are not recorded in CommCare due to patient confidentiality and are only communicated in person to the clinic nurse or HIV counselor.

### National Health Insurance Scheme enrolment

Since the TLI began accepting NHIS reimbursements in 2017, payment using NHIS was used for 77% of female patients receiving tests, 87% of pregnant women, and 42% of male patients (mostly under the age of 5). In Ghana overall, national enrolment in the NHIS was estimated at 40% in 2016, with 60% of the enrolled population being pregnant, under 18, over 70, or in the poorest income bracket, and therefore exempt from paying premiums.^11^ In 2017, close to 70% of all patients at the TLI paid using the NHIS.

## Lessons learned

### Potential for clinical, epidemiological and research benefits

The TLI experience demonstrates how basic laboratory services can be offered in rural, low-resource communities. Access to diagnostics can promote efficient treatment and facilitate appropriate linkage to care.

Additionally, it provides valuable data, revealing disease burdens and health care-seeking trends, which could be critical insights for public health interventions. For example, it is estimated that 2% of babies born in Ghana have sickle cell disease, and that sickle cell trait prevalence may be around 30% nationally.^12^

At the TLI, 12.3% of female patients and 16.97% of male patients tested positive for sickle cell disease or sickle cell trait (the oxidation-reduction method does not distinguish between trait and disease). It is possible that this area of Ashanti has lower rates of sickle cell trait and sickle cell disease, and this demonstrates how local laboratory services can reveal specific trends and inform public health efforts. Secondly, the age demographic served by the TLI is highly skewed toward reproductive age among female patients, and under 5 years among male patients. This demonstrates that mothers bring young boys to the clinic regularly, but adult men visit less commonly. Conversely, female patients are far more likely receive diagnostics after the age of 5, and often during pregnancy. Integration with the NHIS system reveals which demographics are currently best served by national insurance, and provides insight into healthcare-seeking behaviours among these groups. For example, 87% of pregnant women used the NHIS to pay for TLI services, and further analysis could reveal whether they are getting the antenatal tests at the recommended frequency throughout pregnancy. Data from a larger network of local centralised laboratories following the TLI model in rural settings could support research into healthcare needs and disease burdens that are currently very difficult to investigate, and the value of this knowledge to the development of public health programmes has great potential.

### Human resources and technology

The TLI model relies on well-trained laboratory technologists, managers, nurses, and motorbike riders, but the model does not pay for nurses who are government staff. The system fully depends on the dedication and enthusiasm of clinic healthcare workers who, in addition to collecting samples and ordering the tests, must use CommCare to request those tests and view results. Since clinics also retain paper records, using CommCare results in additional, and often redundant, record keeping for already busy clinics. If this model is to be expanded, extra support staff and streamlined record keeping is needed. The TLI laboratory technicians currently on the staff are from a large urban area, and relocating to a rural area was challenging; the TLI provided higher salaries as an incentive. If the model is replicated, staff retention of laboratory technicians in rural areas could be difficult, and staff motivation would need to be a priority.

Besides workload, one factor impacting the services offered at the TLI is that clinic healthcare workers are trained and qualified to order a limited scope of tests, meaning that the TLI can perform tests that clinics do not perform and are not equipped to interpret. These include liver and kidney function, lipid testing, and others. Increased laboratory services at the local level could inform governments on what additional training nurses and midwives could be given in order to take advantage of more tests, expanding the services available at local levels. It is also possible that the TLI could offer testing requested by hospitals for patient monitoring at a local level.

### Context for implementation

Various factors influence when and if building new laboratory systems is appropriate in rural areas of sub-Saharan Africa or other low-resource settings. First, the laboratory system should fill a void in the healthcare system, and not detract from or be redundant with any services already offered locally. For example, if laboratory services are (or are expected to be) offered within clinic facilities, establishing a parallel laboratory would reduce the need for these services to be prioritised. Rural laboratories should be established in locations with no other diagnostic facilities available within a reasonable distance. The TLI laboratory in Ghana works only with facilities where no laboratory services are offered at the clinic, and that do not have alternative facilities within a reasonable distance given the terrain and access to public transport.

Second, laboratories should be established in rural locations with a number of well-attended health facilities. The ideal location for a laboratory is one where enough samples can be collected to result in full-time or nearly full-time utilisation of laboratory staff and equipment – efficiency and cost-effectiveness of the system decreases if only a small number of samples are delivered each day. The location of the TLI was established in a populated rural area where the nearest reliable diagnostic facility was at the district hospital, in most cases tens of kilometres away – ensuring steady demand from the referring health centres. Third, the methods for transporting and storing samples should be considered carefully when designing any rural laboratory system or specimen referral programme. There must be an inexpensive way to transport samples, ideally one that does not require any equipment or technical skill beyond what is already locally available.

Finally, the laboratory system must be enthusiastically supported by local government health departments. Locations of laboratories and the scope of diagnostic services should ultimately be determined by them, and the easy communication of reports and data between facilities and other levels of the health system should be part of the programme’s design.

### Sustainability

The TLI was established using a grant from Becton Dickinson and Company and in-kind donations from GlaxoSmithKline, and is now sustained largely through internally generated funds with continued support from Becton Dickinson. In areas like Ghana with national or health insurance systems, the model has potential to be fully or partially financially sustainable through insurance reimbursements. In 2017, NHIS reimbursements were sufficient to cover 130% of all basic laboratory operation costs (reagents, consumables, office supplies, electric bills, and similar), excluding salaries. Many tests require only low-cost reagents, so services can be inexpensive for patients paying out of pocket. Demand for services at the TLI have consistently increased since the project was launched, and continues to increase, suggesting that the laboratory is not yet meeting the community demand.

Encouragement to healthcare workers from TLI management and Ghana Health Service, as well as adding additional nearby clinics to the TLI network, could substantially increase revenue to the laboratory, bringing it closer to true financial sustainability. Further research should be conducted on business models for laboratories operating in countries without national health insurance, and the TLI model will continue to work toward a business model that can cover all staff salaries.

### Conclusion

Lessons learned are summarised in Box 1. The TLI model demonstrates that a strong national laboratory system covering rural areas has potential for both epidemiologic surveillance and research on new or improved diagnostics. All or many components of a successful laboratory system already exist in rural areas, and there is often no need to invest in costly and complicated infrastructure, or in extensive training if services fit within what health workers are equipped to offer and interpret. Lastly, there is potential for laboratory systems in rural areas to be wholly or partially financially sustainable, and this should be considered a priority for public health programmes worldwide.

Lessons learned

- A strong national laboratory system covering rural areas has potential for both epidemiologic surveillance and research on new improved diagnostics.
- All components already exist to implement a successful diagnostic system. There is no need to invest in costly and complicated infrastructure or training as long as the scope of services fits within the range of tests that health workers are equipped to offer and interpret.
- There is potential for laboratory systems in rural areas to be wholly or partially financially sustainable.
